# Leptospirose révélée par une pancréatite aiguë

**DOI:** 10.11604/pamj.2019.34.83.15680

**Published:** 2019-10-11

**Authors:** Soufiane Diyas, Moussa Lezreg, Soufiane Badsi, Brahim Housni

**Affiliations:** 1 Service de Réanimation, CHU Mohamed VI, Faculté de Médecine et de Pharmacie d’Oujda, Université Mohammed 1er, Oujda, Maroc

**Keywords:** Leptospirose, pancréatite, Maroc, Leptospirosis, pancreatitis, Maroc

## Abstract

La leptospirose est une anthropozoonose ubiquitaire, particulièrement fréquente en zone tropicale sont des bactéries à Gram négatif aérobies strictes de l'ordre des Spirochaetales et du genre Leptospira dont la transmission se fait par l'eau contaminée par les urines des nombreux animaux porteurs. La pancréatite aiguë est une des complications rares associées à la leptospirose avec un taux de mortalité élevé. Nous rapportons le cas d'un patient de 66 ans chez qui le diagnostic d'une leptospirose a été posée suite à une complication rare de la pancréatite. Notre patient était admis dans notre structure dans un tableau de choc septique nécessitant un support hémodynamique et ventilatoire avec recours à une épuration extra rénale; avec bonne évolution clinique.

## Abstract (en)

Leptospirosis is an ubiquitous anthropozoonosis particularly frequent in the tropical climate zone. It is caused by gram negative, strictly aerobic bacteria belonging to the order Spirochaetales and to the family Leptospira whose transmission usually occurs through contact with water sources contaminated with animal urine (carriers). Acute pancreatitis is a rare complication of leptospirosis; it is associated with high mortality rates. We report the case of a 66-year old patient in whom the diagnosis of leptospirosis was made after a rare complication of pancreatitis. Our patient was admitted with septic shock requiring hemodynamic and respiratory support with renal dialysis. The outcome was favorable.

## Introduction

La leptospirose est une anthropozoonose ubiquitaire, particulièrement fréquente en zone tropicale sont des bactéries à Gram négatif aérobies strictes de l'ordre des Spirochaetales et du genre Leptospira. La transmission se fait par l'eau contaminée par les urines des nombreux animaux porteurs et survient préférentiellement sur des personnes exposées (agriculteurs, jardiniers, égoutiers) et chez des sportifs en eau vive. Cliniquement, la leptospirose est responsable de manifestations variées, allant d'un syndrome pseudogrippal bénin à une atteinte hépatorénale potentiellement létale [1]. La pancréatite aiguë est une des complications rares associées à la leptospirose avec un taux de mortalité élevé.

## Patient et observation

Il s'agit d'un patient âgé de 66 ans tabagique chronique suivi pour hyperplasie bénigne de la prostate (HBP) sous alpha bloquant; admis en réanimation pour la prise en charge d'un choc septique. Le patient présentait depuis une semaine: une asthénie, des myalgies diffuses et une hyperthermie à 39°C avec des épigastralgies sans notion de prise médicamenteuse. L'examen clinique notait un ictère cutanéo-muqueux, sans signes d'insuffisance hépatocellulaire. Il était tachycardé à 114 et instable sur le plan hémodynamique avec une tension arterielle (TA) à 64/48. Il avait bénéficié d'un monitorage invasif de la TA et la prise d'une voie veineuse centrale (VVC) jugulaire interne avec un remplissage vasculaire et l'introduction de la noradrénaline a raison de 2 mg/h. Sur le plan respiratoire, le patient présentait une polypnée superficielle, l’auscultation de même que la radiographie étaient sans particularité. L'examen neurologique mettait en évidence un ralentissement idéomoteur sans signe de focalisation. Il n'existait pas de plaie cutanée. Le bilan biologique avait objectivé: créatinine 62 mg/l, urée: 2.45 g/l; natrémie: 135 mEq/l, kaliémie: 4 mEq/l; leucocytes: 27000/mm^3^, Hb: 12.8 gr/dl, plaquettes: 49 000/mm^3^; taux de prothrombine: 73%; lipasémie: 2 508 UI/l; TGO: 99 UI/l, TGP: 99 UI/l, gamma GT: 504 UI/l; phosphatases alcalines: 166 UI/l, bilirubine totale: 179 mg/l; CRP: 318 mg/l; lactate à 1.58; gazométrie une acidose métabolique. Une échographie abdominale retrouvait un pancréas tuméfié hypo-échogène, une vésicule biliaire collabée; sans dilatation des voies biliaires intra- et extra-hépatiques. Un scanner abdominal sans injection était ensuite effectué, retrouvant un pancréas augmenté de volume, avec un épanchement liquidien hépatique. En raison de la dégradation de l'état neurologique, le patient est placé sous ventilation mécanique, avec support hémodynamique par noradrénaline, et il avait bénéficié de 7 séances d’hémodialyse. Sur le plan infectieux, les sérologies virales A, B, C, VIH, étaient négatives. La sérologie de leptospirose, confirmée à 15J d'intervalle. La patiente avait bénéficié d'un traitement antalgique, d'une réhydratation parentérale et d'une antibiothérapie à base de Ceftriaxone 2 G/J pendant dix jours. L'évolution clinico-biologique était favorable.

## Discussion

La leptospirose se transmet habituellement à l'homme à partir d'un réservoir animal, essentiellement le rat, et dépend d'une infection rénale chronique et de l'émission de spirochète virulent du genre leptospira dans l'urine. L'homme est habituellement l’hôte final, la contamination se faisant à l'occasion de baignades dans les rivières, étangs ou gravières, habituellement durant la période estivale. Le temps d'incubation se situe entre 7 et 12 jours, avec des extrêmes de 2 à 20 jours. Cliniquement, la leptospirose est responsable de manifestations variées, allant d'un syndrome pseudo-grippal bénin à une atteinte hépatorénale potentiellement létale. Cette maladie peut se présenter soit sous forme anictérique, qui se voit dans 80 à 90% des cas, ou sous forme ictérique, retrouvée dans 5 à 10% des cas qui est justement la forme vue chez notre patiente et qui est la forme la plus grave. L'association de l'atteinte hépatorénale avec des manifestations hémorragiques définit le syndrome de Weil, de pronostic défavorable [1-3]. La pancréatite aiguë est l'une des complications les plus rares de la leptospirose. Elle peut se manifester par des douleurs abdominales, des nausées, des vomissements, de la diarrhée, de l'anorexie ou encore un syndrome fébrile [2, 4, 5]. Le diagnostic bactériologique est marqué par le faible rendement des cultures (hémocultures, urine). Il repose sur la micro-agglutination qui permet le sérotypage des souches et le titrage des anticorps, mais il doit lui être associé le titrage des IgM par Elisa, plus sensible. La “polymerase chain reaction (PCR)” semble la solution d'avenir pour un diagnostic précoce, permettant alors de réaliser une antibiothérapie plus précoce. Pour le diagnostic de la pancréatite aiguë, le dosage de la lipasémie chez les patients présentant une douleur abdominale est en général suffisant. Notons que l'hyper-amylasémie peut se voir dans la leptospirose due soit à l'insuffisance rénale, ou à d'autres raisons inconnues [6]. Dans notre cas, la lipasémie était très élevée. Une hyper-amylasémie est retrouvée jusque dans 65% des cas de leptospiroses [6]. Une revue de la littérature retrouvait en 1991, 4 cas documentés de pancréatite dans la littérature anglo-saxonne [6]. Depuis, d’autres cas ont été répertoriés. Une série de 17 cas de leptospirose chez des enfants [5] du Pacifique était répertoriée sans plus de précision concernant la pancréatite, qui n'apparaissait que biologique. Aucun examen complémentaire, notamment radiologique, n'était réalisé. D'autres observations chez l'adulte ne peuvent être vraiment retenues comme des pancréatites vraies, en particulier à cause du manque d'explorations morphologiques pancréatiques ou de l'absence d'anomalie pancréatique [7]. Le traitement de la pancréatite aiguë compliquant une leptospirose repose sur l’antibiothérapie contre la leptospirose, la réhydratation intraveineuse et une nutrition parentérale ou entérale et la prévention repose essentiellement sur le contrôle du réservoir animal et hydrique [2, 8]. Dans les zones d'endémies, une pancréatite associée à un syndrome hépato-rénal fébrile fera rechercher le diagnostic de leptospirose, bien qu'exceptionnel.

## Conclusion

L'atteinte pancréatique, documentée par la lipasémie ou la tomodensitométrie reste rare et est possiblement d'origine plurifactorielle, au cours de la leptospirose. Dans notre expérience, elle constitue un facteur de gravité pronostique; l'établissement d'un diagnostic précoce est nécessaire pour une prise en charge thérapeutique rapide permettant ainsi d'éviter une évolution fatale.

## Conflits d’intérêts

Les auteurs ne déclarent aucun conflit d'intérêts.
